# Research on the Glial–Lymphatic System and Its Relationship With Alzheimer’s Disease

**DOI:** 10.3389/fnins.2021.605586

**Published:** 2021-06-16

**Authors:** Danhua Ding, Xinyu Wang, Qianqian Li, Lanjun Li, Jun Wu

**Affiliations:** ^1^Department of Neurology, The First Affiliated Hospital of Zhengzhou University, Zhengzhou, China; ^2^Department of Rheumatology, Peking University Third Hospital, Beijing, China

**Keywords:** glial–lymphatic system, aquaporin 4, β-amyloid, Alzheimer’s disease, brain

## Abstract

Metabolic waste clearance is essential to maintain body homeostasis, in which the lymphatic system plays a vital role. Conversely, in recent years, studies have identified the glial–lymphatic system in the brain, which primarily comprises the inflow of fluid along the para-arterial space. Aquaporin-4 mediates the convection of interstitial fluid in the brain and outflow along the paravenous space. β-Amyloid deposition is a characteristic pathological change in Alzheimer’s disease, and some studies have found that the glial–lymphatic system plays an important role in its clearance. Thus, the glial–lymphatic system may influence Alzheimer’s disease severity and outcome; therefore, this review summarizes the current and available research on the glial–lymphatic system and Alzheimer’s disease.

## Introduction

The clearance of metabolically generated waste helps to maintain homeostasis, wherein the lymphatic system plays a vital role. Recent studies have identified a lymphatic-like pathway in the brain, known as the glial–lymphatic system. This system has been confirmed to play an important role in β-amyloid (Aβ) clearance (Iliff et al., 2012; Tarasoff-Conway et al., 2015); Aβ deposition is characteristic of Alzheimer’s disease (AD). The glial–lymphatic system in the brain may be involved in the occurrence, development, and impact of AD. The discovery of the glial–lymphatic system in the brain and its subsequent research will help to comprehensively understand brain function and in the early identification of AD, allowing early intervention, which can significantly delay or prevent the progression of AD. This paper reviews the current research on the glial–lymphatic system and its influence on AD, including mechanistic studies, diagnosis, and treatment of AD.

## Glial–Lymphatic System

### Research Process of the Glial–Lymphatic System

The cerebrospinal fluid (CSF) plays an important role in the process of removing metabolites from the brain; however, how substances are transferred from the brain parenchyma to the CSF has long been controversial. Rennels et al. (1985) discovered that the CSF entered the parenchyma along the para-arterial space. Their study injected horseradish peroxidase into the subarachnoid space and found that the solute in the CSF can pass through the para-arterial space within minutes. A fluid pathway parallel to the vasculature of the brain parenchyma enters the extracellular space of the entire neural axis. Based on this, the “rapid paravascular circulation of CSF” hypothesis was proposed; that is, the CSF flows into the brain *via* the arterial perivascular spaces and then leaves *via* venous perivascular spaces a few minutes later. Cserr et al. disputed this by microinjecting a large-molecular-weight tracer (colloidal gold/Evans blue or rhodamine-stained ink or albumin) into the perivascular space of arteries or veins on the brain surface or the subarachnoid space of the cerebral cortex of anesthetized rats (Ichimura et al., 1991). *In vivo*, the subsequent distribution was tracked under a microscope, and the histological sections were observed by light and electron microscopes. The flow path confirmed that the perivascular space could be used as a channel for fluid exchange between the brain and CSF, but this flow was observed to be slow and its direction changed unpredictably. Therefore, they questioned the “rapid paravascular circulation of CSF” hypothesis, instead hypothesizing that the “bulk flow of CSF within the perivascular space which is slow and variable in direction.” These differences may be due to multiple factors such as the molecular weight of the reagents used and the experimental design. Considering that glucose metabolites are produced during brain activation, Gerald et al. conducted a series of studies to detect traces of glucose metabolites in the perivascular space and cervical lymph nodes (Gandhi et al., 2009; Ball et al., 2010), providing compelling evidence for the idea that the paravascular space is a pathway to clear metabolic waste. Iliff et al. injected a fluorescent tracer into the cerebellar medulla pool and found that the CSF in the subarachnoid space entered the parenchyma along the para-arterial space and that brain interstitial fluid was cleared along the paravenous space. Further studies found that this fluid flow reduced the rate of tracer clearance by 70% in aquaporin 4 (AQP4) knockout mice (Iliff et al., 2012). This AQP4 clearance pathway, which relies on astrocytes, is functionally similar to the peripheral lymphatic system; thus, it is referred to as the glial–lymphatic system (Iliff et al., 2012).

The glial–lymphatic system is a key component of the central lymphatic system, which undertakes the primary processes of material exchange and drainage between the CSF and the interstitial fluid (ISF) of the brain. Radioactive-labeled Aβ injected into the brain of the mouse striatum showed that Aβ was quickly exited along the paravenous space of the glial–lymphatic system. The radiation intensity in the brain was measured at 15 min, 30 min, and 1 h following Aβ injection and showed that the clearance rate was reduced by 55% in AQP4 knockout mice (Iliff et al., 2012). However, Smith et al. (2017) studied AQP4 knockout mice and did not detect any reduction in the CSF tracer in the brain parenchyma, thus questioning the key role of AQP4 in clearance function. However, Mestre et al. (2018) used a meta-analysis of the results of five studies to show that the tracer influx along with the distribution and clearance of interstitial solutes in animals with AQP4 gene deletion were impaired, supporting the initial findings of AQP4-dependent CSF clearance and further confirming the key role of AQP4 in promoting Aβ clearance. The different results obtained by Smith et al. may be because of differences in anesthesia and other technical details. It has long been thought that the central nervous system (CNS) is completely deficient in conventional lymphatic vessels. The discovery of meningeal lymphatic vessels in 2015 has become another compelling evidence of the existence of central lymphatic systems in the brain (Louveau et al., 2015). Aspelund et al. (2015) further confirmed that the lymphatic network is not only distributed around the venous sinuses but also around the intracranial arteries and cranial nerves, exiting the cranium along the cranial nerve or arteriovenous. Meningeal lymphatic vessels can collect intracranial lymph fluid and drain brain immune cells and fluids into the deep cervical lymph nodes, and their functions are regulated by VEGFR3, just as the peripheral lymphatic vessels. The above studies have confirmed the presence of a clearance pathway similar to the peripheral lymphatic system in the brain, i.e., the glial–lymphatic system relying on the astrocyte and the meningeal lymphatics, which together constitute the lymphatic system in the brain (Ichimura et al., 1991). Solutes such as Aβ are eliminated and flow out of the brain through the meningeal lymphatic vessels to the cervical lymph nodes.

It can be seen from the above studies that people’s understanding of the glial–lymphatic system has gone through a long, tortuous, and controversial process. At the same time, it can be seen from the above multiple experiments that AQP4 plays an important role in the glial–lymphatic system. AQP4 is a member of the aquaporin family. Aquaporin is a transmembrane channel protein that can mediate the transport of water and other small molecular solutes, such as glycerol, ammonia, and some gases (carbon dioxide, nitric oxide, etc.), between cells (Hara-Chikuma and Verkman, 2006; Kitchen et al., 2015b; Ciappelloni et al., 2019). AQP4 is highly expressed in brain tissues and distributed in the astrocyte membrane in polar form. It is the main molecular pathway of the CNS regulating water permeability and has dual regulatory effects: promoting water elimination in vasogenic edema and promoting astrocyte swelling in cytotoxic edema (Galoyan, 2000; Manley et al., 2004). Changes in AQP4 expression and polarity will affect its function (Ciappelloni et al., 2019). AQP4 is increased on the cell surface in a calmodulin-dependent manner: calmodulin binds directly to the terminus of AQP4, causing specific conformational changes that drive the cell surface localization of AQP4 (Kitchen et al., 2015b; Salman et al., 2017; Ciappelloni et al., 2019). It can be seen that the diversity of AQP4’s functions, its cell surface localization mechanism, and its important role in the glial–lymphatic system provide the possibility to regulate AQP4 and then affect the glial–lymphatic system, which may be one of the better ways to clear Aβ.

**FIGURE 1:**
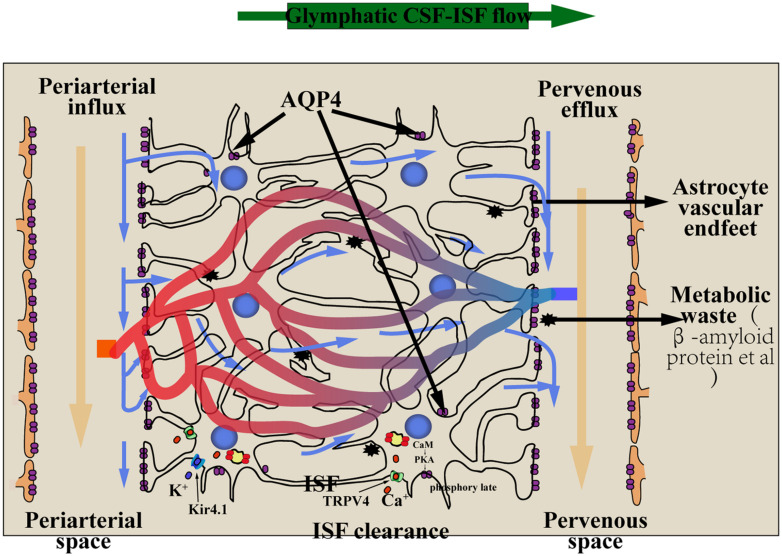
The glial–lymphatic pathway (*blue arrows*) clears metabolic waste from the ISF through the brain parenchyma. It is includes three components: (1) CSF within the subarachnoid and cisternal spaces flows into the brain specifically *via* periarterial spaces; (2) CSF–ISF exchange within the brain parenchyma facilitated by aquaporin-4 (AQP4) water channels that are positioned within perivascular astrocyte end-foot processes; (3) CSF and waste flow into perivenous spaces and exit the cranium through the meningeal lymphatic vessels into the cervical lymph nodes. Nervous system edema involves the translocation of AQP4, the mechanism of AQP4 translocation must be extracellular calcium Ca^2+^, CaM and PKA. The transient receptor potential vanilloid 4 (TRPV4) channel is linked to the pathology of the edema，and influence the subcellular relocalization of AQP4：Extracellular calcium ions can flow into astrocytes through the TRPV4 channel to activate calmodulin (CaM), activating PKA, which phosphorylates AQP4 at Ser276, and AQP4 relocalize to the plasma membrane. Kir4.1 and AQP4 function in coupling with each other to mediate water transport. *AQP4*, aquaporin-4; *CSF*, cerebrospinal fluid; *ISF*, interstitial fluid; *TRPV4*, the transient receptor potential vanilloid 4; *CaM*, calmodulin; *PKA*, protein kinase A; *Kir 4.1*, Inward rectifying potassium channel 4.1.

As described above, in the brain, the CSF is rapidly exchanged with the ISF *via* the glial–lymphatic pathway that comprises three serial elements: a para-arterial CSF influx route, a paravenous ISF efflux route, and an intracellular *trans-*astrocytic path that couples the two extracellular paravascular routes (Figure 1).

#### Driving Force of the Glial–Lymphatic System

The driving force of the glial–lymphatic system is not from a single source, but mainly consists of the flow of the CSF, arterial pulse, cardiac impulse, vasomotor movement, and respiratory movement.

#### Continuous Generation of CSF

The continuously generated CSF of the choroid plexus drives fluid from the ventricular system into the subarachnoid space, which then flows into the interstitial space along the para-arterial space. The exchange of liquids and the removal of metabolites are particularly important (Jessen et al., 2015).

#### Arterial Pulsation

It has been shown that ligation of the unilateral internal carotid artery reduces arterial pulsation by 50% and at the same time reduces the exchange rate of the CSF and ISF (Iliff et al., 2013b). An adrenaline receptor agonist, dopamine, can increase arterial pulsation by 60% while promoting the exchange of paraspinal CSF and ISF. It is speculated that the pulsation of cerebral arteries is also an important driving force for the glial–lymphatic system (Iliff et al., 2013b).

#### Cardiac Impulse

Kyrtsos and Baras showed that an increase in heart rate could reduce the deposition of Aβ, suggesting that heartbeat, which is the origin of arterial pulses, also affects the glial–lymphatic system’s clearance function (Kyrtsos and Baras, 2015). Conversely, an increase in blood vessel stiffness significantly increases Aβ deposition; it may be explained by the fact that blood vessel stiffness weakens the arterial pulse conduction, which consequently reduces the convection of ISF in the brain and ultimately reduces the clearance of the glial–lymphatic system. Kress et al. (2014) found that aging could weaken the circulation of paraspinal CSF, which can be reduced by 27% with arterial pulses, further supporting the above conclusion. That of Hablitz and other studies have found that under anesthesia, the heart rate and lymph fluid inflow are strongly inversely correlated; that is, a decrease in heart rate will accelerate glial–lymphatic transport (Hablitz et al., 2019). This is because anesthetic drugs affect not only the heart rate but also the vasomotor contraction and respiration. The final observed result is the comprehensive effect of anesthetic drugs on glial–lymphatic transport.

#### Vasomotor Movement

Using magnetic resonance, it was found that slow vasomotor motion is also an important driving force for the glial–lymphatic system. Slow vasomotor motion causes lower-frequency pulsations in its unique spatiotemporal mode, which affects the glial–lymphatic system transport (Kiviniemi et al., 2016).

#### Respiratory Movement

It has been found that respiratory movement is also an important driving force for the glial–lymphatic system. Breath-derived pulsations primarily influence the space around the veins and concentrically facilitate the removal of substances along the paravenous space (Kiviniemi et al., 2016). Other studies have found that the level of Aβ in the CSF of patients with obstructive sleep apnea is decreased and that It is very likely that the apnea affects the exchange of CSF and ISF (Ju et al., 2016). The pressure caused by breathing promotes the flow of ISF into the brain, and during apnea, increased intrathoracic pressure, venous dilation, narrowing of the perivenous space, and blockage of the paravenous pathways weaken the outflow of solute in the glial–lymphatic system along the paravenous gap. Simultaneously, repeated pressure fluctuations in patients during apnea also hindered the clearance of metabolites through the glial–lymphatic system (Kiviniemi et al., 2016).

In short, the driving force of the glial–lymphatic system primarily comes from the continuous generation of CSF, arterial pulsation, cardiac impulse, heart rate, the slow vasomotor motion, and the pressure caused by respiratory movement. The related driving forces that regulate the glial–lymphatic system, affecting its fluid exchange, may also affect the removal of harmful substances such as Aβ and tau protein (Iliff et al., 2012; Kriegel et al., 2018), thereby improving the prognosis of AD and making the glial–lymphatic system an important part of the potential therapeutic target for AD. The glial–lymphatic system may be an important part of the potential treatment of AD.

#### Factors Affecting the Function of the Glial–Lymphatic System

##### Sleep

Xie et al. found that the tracer inflow along the para-arterial space increased significantly during sleep, which may be because of the 60% increase in the interstitial space of the brain during sleep, causing a reduction in the tissue’s resistance to fluid flow. Resistance decreases, and the clearance efficiency of the glial–lymphatic system is significantly improved during sleep (Xie et al., 2013), while norepinephrine signaling by the locus coeruleus is essential for wakefulness (Steriade et al., 1993; Carter et al., 2010; Constantinople and Bruno, 2011). Adrenergic receptor inhibitors affect awakening while accelerate the inflow rate of the CSF along the glial–lymphatic channels (Xie et al., 2013). This suggests that the sleep–awakening state rather than the circadian rhythm determines the interstitial space of the brain, thus affecting the clearance efficiency of the glial–lymphatic system (Xie et al., 2013). By applying nuclear magnetic resonance and injecting gadopentetate dimeglumine into the CSF, it was found that the glial–lymphatic system had the highest clearance efficiency in the lateral position compared with the supine and prone positions. The most popular sleeping position (lateral position) is an evolutionary adaptation that optimizes the efficiency of metabolite clearance during sleep (Lee et al., 2015).

The above studies have demonstrated the important role of sleep in the clearance function of the glial–lymphatic system and the maintenance of metabolic homeostasis and that sleep may affect the pathophysiological processes of the development of brain diseases. The effects of clearance function may demonstrate the role of sleep and its underlying mechanism along with the diagnosis and possible treatment options of brain diseases associated with it.

#### Anesthetic Drugs

In recent years, studies have focused on the effects of anesthetic drugs on the function of the glial–lymphatic system and their underlying mechanisms. Dexmedetomidine, a selective α2 agonist that can hyperpolarize locus coeruleus neurons, reduces the release of norepinephrine and thus plays a role in inducing hypnosis (Keating et al., 2012). Benveniste et al. (2017) showed that, compared with isoflurane alone, dexmedetomidine combined with low-dose isoflurane in anesthetized rats had a 32% increase in the transport efficiency of the glial–lymphatic system. This suggests that, in addition to the known effects of anesthesia, i.e., causing a loss of consciousness, these drugs possess specific pharmacological effects, especially narcotic drugs that inhibit noradrenergic function, which significantly influence the glial–lymphatic system transport function (Benveniste et al., 2017). Dexmedetomidine may improve postoperative cognitive function by improving brain metabolite clearance during anesthesia or sedation.

From the above research, it can be seen that the development and the selection of drugs that induce hypnosis and reduce norepinephrine release can enhance the transport function of the glial–lymphatic system and its clearance of metabolites, thereby improving patients’ cognitive function, making it a possible therapeutic approach for dementia-related diseases. Hablitz et al. (2019) have found that different anesthetic drugs can have different effects on the lymphatic drainage rate. This is also important in regulating the glial–lymphatic system drainage rate, thus promoting the elimination of Aβ, tau protein, and other metabolites and improving or delaying AD. This provides wider treatment options for diseases associated with the inner nervous system, including AD.

## Glial–Lymphatic System and AD

Alzheimer’s disease is a degenerative disease of the CNS that is characterized by progressive cognitive impairment and abnormal behavior in the elderly and pre-senile. It is clinically manifested as memory impairment, aphasia, apraxia, frustration, and impaired visual space ability and abstraction (Selkoe, 2013; Attems and Jellinger, 2014). The characteristic pathological changes in AD include senile plaques, neuron fiber tangles, and extensive neuronal loss, while the pathogenesis is not completely clear (Attems and Jellinger, 2014), involving the Aβ cascade hypothesis, the tau protein abnormal modification hypothesis, the cholinergic injury hypothesis, the cholinergic injury hypothesis, etc. (Hardy and Allsop, 1991; Strittmatter et al., 1993; Schorderet, 1995). some studies have been observed in neurodegenerative diseases such as AD, the activation of microglia and the production of inflammatory cytokines (Yin et al., 2016; Bordone and Salman, 2019), mitochondrial dysfunction, breakdown of the tricarboxylic acid cycle, and insufficient energy and oxidative stress (Yin et al., 2016; Bordone and Salman, 2019). These studies suggest that brain energy metabolism, oxidative stress, and neuroinflammation are interrelated and may also jointly participate in or influence the progression of brain aging and the etiology of Alzheimer’s disease (Yin et al., 2016; Kitchen and Salman, 2019). Up to now, among the many theories regarding the pathogenesis of AD, the Aβ cascade hypothesis remains dominant. Many studies have also confirmed that the accumulation of Aβ in the brain leads to neuroinflammatory damage and has toxic effects on neurons and synapses, leading to a series of impairments such as cognitive decline. Aβ plays a vital role in the development of AD (Selkoe, 2013). Previous studies have found that the brain’s clearance of Aβ primarily includes the following: (1) Aβ enzyme degradation—degradation by proteases such as enkephalinase and insulin-degrading enzyme (Yoon and Jo, 2012); (2) cell phagocytosis—Aβ is internalized by the microglia, astrocytes, and neurons and then degraded intracellularly (Mandrekar et al., 2009; Lee and Landreth, 2010; Thal, 2012; Kanekiyo et al., 2013); and (3) blood–brain barrier transport—low-density lipoprotein receptor-associated protein mediates Aβ transport from the brain to peripheral blood circulation (Kanekiyo et al., 2012).

As mentioned earlier, recent studies have shown that the glial–lymphatic system is also involved and affects Aβ clearance. It has been found that the decrease of AQP4 function around the blood vessels, dysfunction of the glial–lymphatic system, and Aβ aggregation seem to form a continuous vicious feedback and cycle that promotes Aβ deposition, ultimately leading to neurodegenerative diseases (Kress et al., 2014). Dysfunction of the glial–lymphatic system increases the Aβ levels not only in the brain parenchyma but also in the blood vessel walls, leading to cerebral amyloid angiopathy (CAA), which can cause thickening of the basement membrane and narrowing of the perivascular cavities, blocking the clearance pathway of the glial–lymphatic system (Roher et al., 2003; Nedergaard, 2013). It can be seen that the decrease of glial–lymphatic system function and Aβ deposition affect the development of CAA, and CAA is also involved in this vicious cycle, thereby accelerating the pathophysiological changes of AD.

Under normal conditions, the production and clearance of Aβ in the body are in a dynamic equilibrium, but when the Aβ metabolism is disordered and the clearance function is impaired, the equilibrium is broken, and Aβ deposition and abnormal accumulation cause a series of damages. Recent studies have found that the glial–lymphatic system also participates in or affects the clearance of Aβ, and its function affects the pathophysiological changes of AD. Related research can be summarized into the following two aspects.

### Animal Studies

Wang et al. (2019) found that blocking the deep cervical lymph node drainage of APP/PS1 double-transgenic AD mice resulted in a significant deterioration of Aβ-related pathophysiology. Peng et al. (2016) found that, in aged AD mice, the amount of CSF flowing into the brain and the clearance efficiency of the cerebral interstitial fluid was reduced along with a significant Aβ deposition, suggesting that the glial–lymphatic system was inhibited. Xu et al. (2015) found that, in AD mice, changes in the expression of AQP4, which plays an important role in the glial–lymphatic system on astrocytes, affect the clearance of endogenous Aβ. AQP4 deficiency can increase Aβ deposition and the risk of cerebral amyloid angiopathy development, which may be explained by the fact that AQP4 deficiency reduces the clearance of Aβ along the paravascular pathway and weakens the activation of astrocytes’ phagocytosis of Aβ. In addition, the study of Kress et al. on C57BL/6 mice found that the function of the glial–lymphatic system in the elderly mice was impaired. Compared with the young mice, the clearance rate of Aβ was reduced by 40%, and the loss of AQP4 polarity was also significantly related to the impaired exchange of CSF to ISF besides the blood vessels. Suggesting that the loss of AQP4 polarity may affect the ISF exchange between CSF and ICF and, thus, affect the clearance of Aβ (Kress et al., 2014) *In vivo* micro-dialysis studies in wild-type mice have shown that the Aβ content of the interstitial fluid in the brain during sleep is lower than that in the arousal period (Kang et al., 2009), suggesting that the glial–lymphatic system may increase Aβ clearance during sleep. Iliff et al. (2013a) demonstrated the exchange of CSF to ISF in 3D imaging of living mice using two different molecular weight contrast agents by enhanced nuclear magnetic resonance. These studies have shown that the glial–lymphatic system exists in the brain, and its clearance effect on Aβ affects the pathophysiological changes of AD animals. Aging and changes in the expression of AQP4 affect the ability of the glial–lymphatic system to clear Aβ, in short, the glial–lymphoid. The function of the system and its related influencing factors may jointly participate in and affect the disease process of AD. The above shows that some factors that affect the clearance of Aβ by the glial–lymphatic system, such as aging and changes in the AQP4 expression, may influence the progression of AD.

### Human Studies

Eide and Ringstad injected gadolinium into the sheath of a female subject and found that it was distributed in the brain and spinal cord by conventional NMR imaging, which indicated that gadolinium in the cerebrospinal fluid could freely convert from cerebrospinal fluid to stromal fluid. The presence of the glial–lymphatic system in the human brain has been confirmed by imaging methods (Eide and Ringstad, 2015). Previous studies have shown that the CSF enters the cerebral parenchyma along the para-arterial space and is cleared along the paravenous space, indicating that the paravascular space is where the CSF and brain parenchyma are exchanged. Recently, another study that evaluated the diffusion tensor image analysis technology in patients with AD found that the diffusivity of water molecules in the paravascular space was positively correlated with the simple mental state assessment scale (MMSE) score, suggesting that the incidence of AD is associated with the glial–lymphatic system (Taoka et al., 2017). Previous studies on the driving force of the glial lymphatic system suggest that a lower CSF pressure impairs the clearance function of the glial lymphatic system for substances such as Aβ, while Tommaso et al. found that AD patients have a low level of CSF compared with the control group and that the level of Aβ in the CSF is directly proportional to the CSF pressure, suggesting a close relationship between the glial lymphatic system and Aβ clearance and its association with the pathogenesis of AD (Schirinzi et al., 2017). Studies have also shown that AQP4 expression in astrocytes plays an important role in the clearance of Aβ and other substances by the glial–lymphatic system. The latest research also found that the age-related change in AQP4 polarity distribution (change in AQP4 localization around blood vessels) is accompanied by an increased deposition of Aβ, suggesting that the change in AQP4 polarity distribution weakens the glial–lymphatic function of AD patients and increases Aβ deposition, thus promoting protein aggregation and neurodegeneration (Zeppenfeld et al., 2017; Kitchen et al., 2020). Other studies have reported an association between AQP4 single nucleotide polymorphisms and the progression of cognitive decline in AD patients, of which rs9951307 and rs3875089 can lead to chronic progressive cognitive decline and rs3763040 and rs3763043 can lead to the rapid progression of cognitive decline, suggesting that the AQP4 gene product affects the cognitive decline in AD patients (Burfeind et al., 2017). The latest research found that carrying the AQP4 rs72878776 and rs491148 minor alleles was associated with a higher Aβ deposition and a shorter sleep duration; that is, genetic variations of AQP4 influence the association between sleep and brain Aβ deposition (Rainey-Smith et al., 2018). However, the full effect of AQP4 single nucleotide polymorphisms on the clearance of Aβ by the glial–lymphatic system and the specific underlying molecular mechanism warrant further investigation. These new evidences further suggest that the glial–lymphatic system that relies on AQP4 expression in astrocytes affects the pathogenesis and progression of AD.

In brief, the above animal and human studies have directly or indirectly demonstrated that the glial–lymphatic system plays an important role in the pathogenesis of AD. The glial–lymphatic system is likely to be an important link for both studying the pathogenesis of AD and as a potential and novel therapeutic strategy. This conclusion, however, warrants further conclusive investigation, along with a more in-depth research to confirm and clarify the specific efficacy and role of the glial–lymphatic system in patients with AD. Although the current research may not be sufficient, it, nonetheless, provides a direction for future studies, which will significantly improve the diagnosis of AD and help us to understand its pathogenesis and design better treatments and may provide insights into other neurodegenerative disorders.

## Risk Factors for Glial–Lymphatic System and AD

### Chronic Traumatic Encephalopathy

Brain trauma is one of the risk factors for the early development of AD (Nemetz et al., 1999; Smith et al., 2013). Studies have found that glial–lymphatic dysfunction appears after brain trauma, and AQP4 gene knockout further aggravates glial–lymphatic dysfunction post-brain trauma (Iliff et al., 2014), which is consistent with the effect of AQP4 on the glial–lymphatic system discovered in 2012 (Iliff et al., 2012).

In cases of mild chronic traumatic encephalopathy, neurofibrillary tangles can be seen in the locus coeruleus (McKee et al., 2016). The locus coeruleus is involved in the maintenance of the sleep–awakening state (Samuels and Szabadi, 2008) and can be affected by brain trauma (Fujinaka et al., 2003). In theory, the structure of locus coeruleus inhibition can reduce the release of norepinephrine and cause cell intercellular space expansion and therefore increase the clearance of proteins through the glial–lymphatic system. In fact, the function of the glial-lymphatic system decreases, possibly due to the more complex network-based neural inhibition and excitation patterns in the locus coeruleus and the norepinephrine system following the loss of neurons in the locus coeruleus. For instance, the lateral preoptic nucleus of the hypothalamus has an inhibitory effect on the locus coeruleus, promoting the sleep state (Chou et al., 2002). Injury of the inhibitory interneurons in the locus ceruleus, the area of white matter between the locus ceruleus and the lateral preoptic nucleus, and/or in the locus ceruleus, which is responsible for the communication with the lateral preoptic nucleus, can increase the tonic activity of the locus ceruleus and reduce the sleep time required for the glial–lymphatic system to function after injury (Chou et al., 2002). Therefore, damage to the locus coeruleus does not necessarily mean the expansion of the interstitial space, and it is possible that the damage of the inhibitory interneurons in the locus coeruleus may lead to the decrease of fluid flow potential in the glial–lymphatic system and affect its function. Not only does Aβ clearance decrease when the glial–lymphatic system function is impaired, resulting in increased Aβ deposition, but studies have also found that chronic traumatic encephalopathy also has phosphorylated tau protein deposition around the cortical sulcular vessels and in astrocytes (McKee et al., 2016). Since the glial–lymphatic system depends on the function of astrocytes and the movement of CSF in the perivascular spaces, the accumulation of proteins such as Aβ and tau in these spaces after traumatic brain injury may lead to the weakening of the glymphatic function and the resulting vicious cycle.

In conclusion, the impaired function of the glial–lymphatic system after traumatic brain injury may affect the progression of traumatic brain injury and thus increase the risk of AD.

### Normal Pressure Hydrocephalus

Normal pressure hydrocephalus is also one of the risk factors of AD (Kang et al., 2018; Ates Bulut et al., 2019). Studies on patients with idiopathic normal pressure hydrocephalus have found that the distribution of CSF along the perivascular space into the brain parenchyma is slower and angiographic than that in the control group (Ringstad et al., 2017). Moreover, the clearance rate slowed down, an enlarged perivascular space was observed using magnetic resonance imaging (MRI) (Tarnaris et al., 2012), and the pathological biopsy revealed abnormal changes in the distribution of AQP4 around the blood vessels (Eide and Hansson, 2018) involved in the pathogenesis of idiopathic normal hydrocephalus. Biopsy of patients with dementia symptoms found that 75% had pathological manifestations of AD (Golomb et al., 2000), suggesting that glial–lymphatic dysfunction is a comorbid phenomenon of AD and idiopathic normal hydrocephalus.

A further in-depth research will provide information on the diagnosis and treatment of these two diseases and their internal connections.

### Diabetes

Diabetes is also a risk factor of AD (Ninomiya, 2019). Conversely, patients with AD have abnormal blood glucose or diabetes by a factor or more than 80% (Janson et al., 2004). Type I is a diabetes-related insulin deficiency and can occur in the brain of AD patients, while type II is a diabetes-related insulin resistance and other diabetes-related indicator abnormalities, such as abnormal end-glycosylation products and insulin-degrading enzyme abnormalities (Yamagishi et al., 2005; Lester-Coll et al., 2006; Frisardi et al., 2010; Jolivalt et al., 2010; Kochkina et al., 2015).

In recent years, research on the glial–lymphatic system has also found a close association between AD and diabetes. Jiang et al. (2017) injected a contrast agent into the cistern of rats and found, using MRI, that the CSF flows into the brain along the para-arterial space and out along the para-superior sagittal sinus space. The presence of contrast agents in the hippocampus and hypothalamus of diabetic rats suggests impaired lymphatic function, and the residual strength of the contrast agents is highly correlated with a diabetes-induced cognitive decline. Studies have shown that mouse diabetes models can present Alzheimer-like pathological manifestations such as Aβ deposition in the brain (Mehla et al., 2014) and that patients with diabetes have a higher risk of AD than do non-diabetic individuals (Baglietto-Vargas et al., 2016). Glial–lymphatic system dysfunction aggravates the deposition of Aβ and other metabolites in the brain, especially in the hippocampus, which may be a potential mechanism of cognitive impairment and a higher risk of AD caused by diabetes mellitus. In addition, vascular disease often occurs in patients with diabetes, with the expansion of the space around the blood vessels (Doubal et al., 2010). The space around the blood vessels is an important part of the glial–lymphatic system, and its expansion may exacerbate the decline of the glial–lymphatic system transport function, or diabetes. The resulting weakened lymphoid function can expand the space around the blood vessels. Therefore, further research on the glial–lymphatic system to clarify the diabetic glial–lymphatic system damage and its impact on cognitive dysfunction is warranted, which is yet another avenue for treatment measures.

Studies over the years have confirmed that islet cells in the pancreas of diabetic patients also have Aβ deposits and that Langerhans cells have phosphorylated tau protein (Miklossy et al., 2010; Halmos and Suba, 2016). A study found that the stabilizing power of amyloid fibrils is associated with AD and type II diabetes (Davidson et al., 2018), further supporting the close association between AD and diabetes. Experts refer to AD as type III diabetes or cerebral diabetes (Steen et al., 2005; Lester-Coll et al., 2006; de la Monte, 2014). Although this view is controversial, it is undeniable that there are inextricable links between AD and diabetes. The above research also shows that a comprehensive and in-depth study of the role of the glial–lymphatic system in the pathogenesis of AD and diabetes will provide more evidence of the association between the two.

### Periodontitis

Recently, Dr. Stephen Dominy’s study found that *Porphyromonas gingivalis*, which causes periodontitis, can cause AD (Dominy et al., 2019). Previous studies have shown that periodontal disease, tooth loss, and AD are strongly associated with each other. *P. gingivalis* has been considered a high risk factor of Aβ deposition and even AD (Noble et al., 2009; Kamer et al., 2015). A study also found that men with clinical diagnosis of mild to moderate AD have periodontitis associated with significant cognitive decline (Ide et al., 2016). Dental interventions were performed on patients (having gingivitis/periodontitis) with a clinical diagnosis of mild AD, where improved memory was observed in more than 50% of the participants (Rolim Tde et al., 2014). The specific mechanism by which periodontal disease increases the risk of AD has not been elucidated (Harding et al., 2017). Some studies have explored the mechanism through animal models, and the results suggest that the possible mechanism of periodontal disease increasing the risk of AD is that *P. gingivalis* transferred to the brain of mice changes the circadian activity of the microglia, leading to disordered sleep patterns in mice (Takayama et al., 2016). This may affect the function of the glial–lymphatic system and increase the deposition of Aβ; that is, periodontal disease increases the deposition of Aβ by triggering glial–lymphatic system dysfunction, which further shows the association between the glial–lymphatic system and the pathogenesis of AD.

### Primary Open-Angle Glaucoma

Some studies have found that primary open-angle glaucoma is associated with AD. The prevalence of glaucoma is 25.9% compared with 5.2% in the control group (age-matched inpatients without AD) (Bayer et al., 2002). Aβ co-localizes with apoptotic retinal ganglion cells in a rat model of chronic ocular hypertension (Guo et al., 2007). The layers of the optic nerve are continuous with the corresponding structures in the skull, and the space around the optic nerve is a continuation of the intracranial subarachnoid space, which is the anatomical basis of the glial–lymphatic system around the optic nerve (Wostyn et al., 2015). The latest research using immunofluorescence staining methods showed that the CSF enters the optic nerve through the space around the blood vessels and is adjacent to the astrocytes at the end of the foot, confirming the presence of the glial–lymphatic system around the optic nerve (Mathieu et al., 2017). Other studies have found that patients with primary open-angle glaucoma have low intracranial pressure, which reduces the fluid flow from the extravascular space of the optic nerve. The fluid in the glial–lymphatic system may stagnate at this location and may result in Aβ deposits, thus causing glaucomatous optic neuropathy (McCulley et al., 2015). In short, the high prevalence of glaucoma in AD patients, the similarity in the anatomical basis, and Aβ deposition along with other pathological changes suggest that glial–lymphatic system dysfunction may be one of the common pathogeneses of the two.

## Significance of the Glial–Lymphatic System in the Diagnosis and Progression of AD and the Evaluation of Its Efficacy

Through *in vivo* imaging of mice, Iliff et al. (2013a) demonstrated the exchange of cerebrospinal fluid to interstitial fluid by enhanced nuclear magnetic resonance. Eide and Ringstad performed NMR imaging after intrafascial injection of gadolinium in a female subject. The presence of the glial–lymphatic system in the human brain has been confirmed by imaging methods (Eide and Ringstad, 2015). Since Aβ plays an important role in the pathogenesis of AD, the dysfunction of Aβ clearance of the glial–lymphatic system may be one of the inducing factors of AD. Therefore, assessment of the clearance of the glial–lymphatic system can be used as a new method to identifying the risk factors of AD and assessing its progression. If the glial–lymphatic system can be used as an important link or target in the treatment of AD in the future, glial–lymphatic system imaging technology can be used to evaluate therapeutic effects. In conclusion, imaging and dynamic monitoring of the glymphatic system is of great significance in the diagnosis and evaluation of the therapeutic effect of AD and has better application prospects.

## Prospects

Although the Aβ theory remains the widely accepted theory regarding the pathogenesis of AD, clinical trials that have cleared Aβ as a single therapeutic target have faced difficulties (Sterner et al., 2016), indicating that there are other factors influencing the pathogenesis of AD. The glial–lymphatic system in the brain is likely to be a link in the pathogenesis of AD. The discovery of the glial–lymphatic system, which continues to unfold after many years of research, in the brain improves our understanding of the anatomical structure of the brain, opening the door to more comprehensive and in-depth research on the physiological functions of the brain and its associated disorders with complicated mechanisms, such as AD.

Professor Maiken Nedergaard recently published the article *CSF Influx Drives Acute Ischemic Tissue Swelling* in March 2020, describing the intrinsic relationship between the glial–lymphatic system and brain edema (Mestre et al., 2020). The glial–lymphatic system is likely to play an unknown but critical role in the pathogenesis of many CNS diseases including AD.

AQP4 has multiple functions (Kitchen et al., 2015a) and has been validated as an important drug target, but no such targeted drug has been approved for clinical treatment (Verkman et al., 2014; Abir-Awan and Kitchen, 2019). AQP4 is an important part of the glial–lymphatic system and plays an important role in the clearance of Aβ. It is highly likely to become a regulatory target of the glial–lymphatic system and thus become a potential target for AD treatment. Kitchen et al. (2020) effectively alleviated brain and spinal cord edema through a targeted regulation of AQP4 expression, which provided enlightenment and experience for the regulation of the brain glial–lymphoid system and also provided new means and methods for the further study of the relationship between the glial–lymphoid system and AD.

In recent years, bioengineering and laboratory technologies such as organ-ona-chip, organoid, and humanized *in vitro* models, especially cutting-edge bioengineering technologies such as high throughput microfluidic device combining lab-on-a-chip technology with 3D organotypic culture, have been used in the study of diseases (Wevers et al., 2018; Raimondi et al., 2019; Salman et al., 2020) and added better research techniques for neurodegenerative diseases such as AD. It is envisaged to apply these technologies to the study of the internal relationship between the glial–lymphatic system in the brain and the pathogenesis of AD. There will be more gratifying discoveries. Over time, as the relevant mysteries are revealed, the diagnosis and treatment of AD that have long plagued humans will be solved.

In short, although the current research on the relationship between the glial–lymphatic system and AD is still in its infancy, studies have provided new ideas and enlightenment for the study of the mechanism of AD and its treatment. We hope that more and more experts and scholars will pay attention to this direction and field, and we look forward to more and better discoveries and breakthroughs in the near future.

## Author Contributions

DD and XW drafted the manuscript and modified the text. DD participated in searching the literature and the revision of articles. JW repeatedly modified the text structure and details. QL and LL participated in the literature review and discussion about article writing and revision. All authors revised and approved the final version of the manuscript.

## Conflict of Interest

The authors declare that the research was conducted in the absence of any commercial or financial relationships that could be construed as a potential conflict of interest.
